# Effects of vitrified cryopreservation duration on IVF and neonatal outcomes

**DOI:** 10.1186/s13048-022-01035-8

**Published:** 2022-09-08

**Authors:** Yuling Mao, Ni Tang, Yanfen Luo, Ping Yin, Lei Li

**Affiliations:** 1 Department of Obstetrics and Gynecology, Center for Reproductive Medicine, Guangdong Provincial Key Laboratory of Major Obstetric Diseases, The Third Affiliated Hospital of Guangzhou Medical University, Guangzhou, China; 2 Key Laboratory for Reproductive Medicine of Guangdong Province, The Third Affiliated Hospital of Guangzhou Medical University, Guangzhou, China; 3 Department of Medicine Laboratory, The Second Affiliated Hospital of Guangzhou University of Chinese Medicine, Guangzhou, China; 4 Department of Medicine Laboratory, Guangdong Provincial Hospital of Chinese Medicine, Guangzhou, China; 5 Department of Clinical Laboratory, The Third Affiliated Hospital of Guangzhou Medical University, Guangzhou, China

**Keywords:** Embryo cryopreservation, In vitro fertilization, Embryo survival, Neonatal outcomes, Storage time, Pregnancy

## Abstract

**Background:**

In this study, we aimed to evaluate the impact of the duration of cryopreservation storage on embryo viability, implantation competence, pregnancy outcome and neonatal outcomes.

**Methods:**

We retrospectively evaluated the outcomes of patients who underwent IVF with vitrified cryopreserved embryos between January 2004 and August 2019 by following the first frozen embryo transfer cycles within the study period. A total of 31,143 patients met the inclusion criteria and were grouped according to the embryo storage time as follows: Group 1 (*n* = 20,926),1–90 days; Group 2 (*n* = 6,472), 91–180 days; Group 3 (*n* = 2,237), 181–365 days; Group 4 (*n* = 746), 366–730 days; and Group 5 (*n* = 762), > 731 days.

**Results:**

The embryo survival rate decreased significantly with longer durations of cryopreservation. The highest and lowest survival rate was recorded in Group 1 and Group 5, respectively (34853/35338; 98.63% vs. 1281/1801; 71.13%; *P* < 0.01). The human chorionic gonadotropin (HCG) detection and clinical pregnancy rate was highest in Group 1 (57.85% and 55. 26%, respectively; *P* < 0.01). Short-term cryopreservation (≤ 3 months) is associated with higher rates of clinical pregnancy. There were no significant differences in neonatal birth weight, neonatal height and congenital anomalies among the groups (*P* > 0. 05).

**Conclusion:**

The prolonged storage time of vitrified embryos negatively affected survival rate and clinical pregnancy rate. It did not have a significant influence on neonatal health. This study provides new findings about the relationship between prolonged storage time of vitrified embryos and clinical outcomes and offers evidence for the safety of using long-stored embryos after vitrification.

## Introduction

In the application of assisted reproductive technology (ART), cryopreservation enables better usage of excess embryos. During cryopreservation, there are various kinds of damage risks, including mechanical damage caused by ice crystal formation and permeable injury as cryopreservation agent and cold shock injury due to cooling [1–3].

Slow-freezing (SF) and vitrification (VF) are commonly used during embryo cryopreservation. SF comprises the use of a programmable machine that decreases the temperature in a controlled fashion; in contrast, VF is used for rapid cooling [4, 5]. In 1985, Rei et al. [6] developed the innovative VF approach by using non-constant high concentrations of cryoprotectant and rapid temperature changes to minimize ice damage. VF is achieved by increasing viscosity together with rapid cooling, thereby allowing the maintenance of solutions at a low temperature without ice crystal formation [7]. In recent years, refrigerant formulations and freezing technologies have been improved and optimized, and VF has replaced SF as the most suitable technique for cellular cryopreservation.

In contrast to SF, VF allows rapid cryopreservation and the solidification of cell(s) and the extracellular milieu into a glass-like state [5, 8]. In doing so, VF improves embryo survival, clinical pregnancy, and live birth rates [9, 10]. However, the high concentrations of dimethyl sulfoxide (DMSO) used during VF may be toxic to the embryo [11–13]. Further, whether exposure to liquid nitrogen during long-term storage affects embryonic survival and development after thawing remains unknown. Therefore, the purpose of this study was to investigate the effects of storage time after VF on the outcomes of freezing–thawing embryo transplantation, and to provide a better reference for clinical work.

## Materials and methods

### Study design and participants

The data were collected from the Center for Reproductive Medicine at The Third Affiliated Hospital of Guangzhou Medical University in Guangzhou, China. We retrospectively reviewed patient and neonatal outcomes following the first frozen embryo transfer (FET) cycles during in vitro fertilization (IVF) with vitrified cryopreserved embryos. The study period was from January 2004 to August 2019. The inclusion criteria were as follows: embryos cryopreserved by VF, procedures corresponding with the first frozen embryo transfer (FET) cycle, and availability of complete follow-up information (including situation of HCG, clinical pregnancy, multiple pregnancy, live birth, ectopic pregnancy, the fetus gender, birth height, birth weight, congenital anomalies). There were no restrictions on age or BMI. There was no donor oocytes cycle in this study. Participants were grouped according to the embryo storage time as follows: Group 1 (*n* = 20,926),1–90 days; Group 2 (*n* = 6,472), 91–180 days; Group 3 (*n* = 2,237), 181–365 days; Group 4 (*n* = 746), 366–730 days; and Group 5 (*n* = 762), > 731 days. Patients with slow thawing, non-first thaw and incomplete information were excluded. Women were excluded if they underwent PGT. The study design was approved by the ethics committee of the Third Affiliated Hospital of Guangzhou Medical University, and written informed consent was obtained from each participant.

### ART procedures

Women were monitored and managed according to the hospital’s clinical protocols. Various controlled ovarian stimulation (COS) protocols were used, with 150–450 IU/day of recombinant FSH or human menopausal gonadotropin in a gonadotropin-releasing hormone antagonist protocol, a long agonist protocol, or a short agonist protocol. The protocols were determined according to each patient's characteristics (age, body mass index (BMI), AFC and AMH). Transvaginal oocyte retrieval was scheduled 35–36 h after HCG injection. ART was performed per standard operating procedure of the hospital.

Conventional IVF or intracytoplasmic sperm injection was performed depending on the semen characteristics and history of previous fertilization attempts. For conventional IVF, cumulus-oocyte complexes were inseminated with progressively motile spermatozoa in fertilization culture medium (G-IVF PLUS, Vitrolife, Gothenburg, Sweden). For intracytoplasmic sperm injection, oocytes were denuded 2–3 h after ovum pickup, and sperm microinjection was performed 5–6 h after oocyte retrieval. Fertilization was checked approximately 16 h after insemination/injection and was determined by the presence of two pronuclei. Embryos were placed in the incubator (K-MINC-1000, Cook Medical, Bloomington, IN, USA) and cultured at 6% CO_2_, 5% O_2_, and 37 °C. G-1 PLUS and G-2 PLUS (Vitrolife, Gothenburg, Sweden) was used for culturing cleavage-stage and blastocyst-stage embryos, respectively. The quality of cleavage-stage embryos was assessed approximately 68 h after insemination/injection, and the quality of blastocyst-stage embryos was evaluated approximately 116 h or 140 h after insemination/injection.

Good-quality cleavage-stage embryos were defined as those with 7–9 symmetrical blastomeres without obvious fragmentation [14]. Good-quality blastocysts were defined as those having reached at least grade 3 expansion and grade A or B for the inner-cell mass and trophectoderm parameters. Poor-quality cleavage-stage embryos were defined as those with blastomere numbers greater than four, fragmentation < 30%, and positive size differences. Poor-quality blastocysts were defined as those having reached at least grade 3 expansion but having grade C inner-cell mass or trophectoderm parameters [15].

Fertilized oocytes were cultured and observed for three days after oocyte retrieval. The embryos destined for freezing were selected according to the embryonic stage and embryo count. For all embryo freezing procedures, 1–2 cleavage-stage embryo VF was performed if embryos were available. If not, blastocyst culture was performed and blastocysts are frozen if available. In cases of fresh ET, when the number of day3 available embryos ≥ 5, blastocysts were cultured and perform fresh ET at the blastocyst stage. When fewer than five embryos are available on day3, the fresh ET is performed at the cleavage stage.

The luteal phase was supported by vaginal administration of micronized progesterone (400 mg/d) initiated on the day of ovarian puncture.

### Embryo cryopreservation techniques and thawing protocols

The embryos were frozen and thawed in accordance with the protocols for the Vitrification Kit (Kato Corp., Shizuoka, Japan). Initially, the embryos were exposed to an equilibration solution (ES)for 5 min at room temperature (cleavage-stage embryo) or 2 min at 37 °C (blastocyst). The embryos were then transferred into a vitrification solution (VS) and incubated for 45 s. Finally, the embryos were set on a Cryotop strip (Kitazato Corp., Japan) in a small volume and immediately plunged into liquid nitrogen. Each storage container contained between one and two embryos. The embryologists have been well trained to perform vitrification technically. The embryos were stored at a constant temperature of -196 °C in the liquid phase of liquid nitrogen tank (Taylor Wharton HC35, Theodore, AL, USA). The level of liquid nitrogen was kept under constant manual surveillance by experienced embryologists to prevent suboptimal storage conditions, and the liquid nitrogen tank was manually refilled twice a week.

Vitrified embryos were thawed by a rapid thawing method on the morning of embryo transfer. For the thawing process of vitrified embryos, the embryos unloaded from the carriers were immediately submerged into the thawing solution for 1 min at 37 °C. Then, the embryos were transferred into the diluent thawing solution for 3 min at room temperature. At the final step, the embryos were moved to the wash solution twice for 3 min at room temperature. After that, the embryos were cultured in culture medium at 37 °C under the gas phase of 5% CO_2_ and 5% O_2_ in an incubator until transfer. If the embryo demonstrated at least 70% of intact cell (up to 30% degraded cell), it was considered viable and was transferred. Embryos showing less than 70% of essential cells, were considered non-viable and were discarded [16].

The laboratory procedures and cryopreservation protocols remained unchanged throughout the study period. The same storage tanks and pieces of technical equipment were used over the years included in the study period. In addition, the clinical freeze-all indication has not changed too.

### Endometrial preparation for FET cycle, and embryo transfer

Endometrial preparation for FET cycle in this study was achieved by natural cycle (NC) or hormone replace treatment (HRT) programs. The ovulation in NC program was determined by monitoring follicular development with transvaginal ultrasonography and hormone levels. The patients in HRT-FET cycles were treated with daily oral estradiol valerate tablets (Progynova, Bayer, Germany) since the second to fourth day of menstruation. When the endometrial thickness reached 7 mm or thicker, 40 mg/day progesterone was intramuscularly administered daily. All embryos transfer treatments were performed on the 4th (cleavage-stage embryo)/6th (blastocyst) day of progesterone exposure or 3(cleavage-stage embryo)/5(blastocyst) days after ovulation using a soft-tipped Wallace (PortexLed., Hythe, United Kingdom) catheter under ultrasound guidance. All patients received luteal support with progesterone after embryo transfer. If transvaginal ultrasound showed gestational sac and embryonic heartbeat 4–6 weeks after embryo transfer, luteal support was continued until 10 weeks of gestational age.

### Outcome measure

The primary outcomes of this study included embryo survival rate, live birth rate and occurrence of congenital conditions. Secondary outcome variable included implantation rate, positive HCG rate, clinical pregnancy rate, multiple pregnancy rate, ectopic pregnancy rate, sex ratio (male/female), birth weight and birth height. Clinical pregnancy was defined as the detection of gestational sacs by transvaginal ultrasound 28 days after ET. The implantation rate was defined as the number of observed gestational sacs divided by the number of transferred embryos. The clinical pregnancy rate was calculated as the number of clinical pregnancies divided by the number of patients. Live birth was defined as the delivery of any viable neonate with a gestational age of 28 weeks or older. Low and high birth weights were defined as birth weights < 2500 g or > 4000 g, respectively.

### Statistical analysis

The statistical analysis was performed using the Statistical Package for Social Science (SPSS) version 22.0. Descriptive data are presented as mean with 1 SD. The differences between groups were tested using the ANOVA test for continuous variables and the Pearson’s chisquare test for categorical variables. In addition, storage group was included as a categorical variable, and Group 1 was used as the reference. We select indicators with statistical differences from ANOVA results and include them in multivariate regression analysis. Multivariable logistic regression was performed to explore the effect of storage time on pregnancy outcome or neonatal outcome after controlling for potential confounders. *P* < 0.05 was considered statistically significant.

## Results

A total of 31,143 first FET cycles met the inclusion criteria. Among the included patients, 20,926, 6,472, 2,237, 746, and 762 patients were classified into Group 1, 2, 3, 4, and 5, respectively.

The patients’ demographics and embryonic cycle characteristics are presented in Table 1. The age range of patients at the time of embryo freezing was 27–40 years. Embryo storage time ranged from 30 to 1,660 days. The female's age at the time of freezing was statistically older in group 3 compared with group 1(*P* < 0.05), but there was no statistical difference between other groups and group 1(*P* > 0.05). While maternal age at ET increased with storage time (*P* < 0.001). The proportion of primary infertility was lowest in group 2 and no difference was seen in the other four storage time groups. The main infertility causes were female infertility, followed by multiple infertility. The proportion of unexplained infertility was gradually reducing with the extended storage time. No between-group differences in stimulation protocols, fertilization method, BMI, endometrial preparation program and numbers of transferred embryos were identified. More patients underwent good-quality embryo and blastocyst transfer in Groups 1 and 2 than in Groups 3–5.

**Table 1:** Demographic and cycle characteristics of FETs stratified by the storage time

	1	2	3	4	5	*P*1	*P*2	*P*3	*P*4
Storage time (days)	1–90	91–180	181–365	366–730	> 731				
Storage time (days), mean ± SD	49.36 ± 18.96	122.335 ± 24.63	244.44 ± 48.93	477.78 ± 98.65	1202.82 ± 457.61				
Number of FET cycles	20,926	6472	2237	746	762				
Maternal age at the time of vitrification (years), mean ± SD	32.38 ± 5.01	33.32 ± 4.05	34.22 ± 3.12	33.27 ± 5.89	32.16 ± 4.92	0.055	0.039	0.068	0.251
Maternal age at the time of thawing(years), mean ± SD	32.44 ± 5.09	33.89 ± 5.23	35.01 ± 5.37	35.00 ± 5.04	35.33 ± 4.89	< 0.001	< 0.001	< 0.001	< 0.001
BMI(Body Mass Index,kg/m^2^	22.06 ± 3.42	21.86 ± 3.38	22.31 ± 3.22	22.17 ± 3.25	22.24 ± 3.40	0.125	0.261	0.149	0.111
Stimulation protocols						0.089	0.052	0.675	0.047
No of receiving long agonist protocol, n (%)	6075(29.03)	1854(28.66)	700(31.32)	225(30.21)	247(32.44)				
No of receiving antagonist protocol, n (%)	11,210(53.57)	3503(54.13)	1084(48.45)	358(48.01)	344(45.11)				
No of receiving short agonist protocol, n (%)	3641(17.40)	1115(17.21)	453(20.23)	163(21.78)	171(22.45)				
Fertilization method, n (%)						0.081	0.661	0.098	0.323
IVF	13,601(65.01)	4294(66.34)	1467(65.59)	493(66.12)	494(64.88)				
ICSI	4608(22.02)	1496(23.11)	504(22.55)	162(21.67)	182(23.87)				
IVF + ICSI	2717(12.97)	682(10.56)	266(11.86)	91(12.21)	86(11.25)				
Endometrial preparation program, n (%)						0.391	0.483	0.552	0.467
Natural cycle	5537(26.46)	1736(26.83)	599(26.77)	194(26.02)	199(26.15)				
Hormonal replacement cycle	15,389(73.54)	4736(73.17)	1638(73.23)	552(73.98)	563(73.85)				
Type of infertility, n (%)						0.037	0.728	0.568	0.666
Primary infertility	10,283(49.14)	3050(47.13)	1109(49.58)	370(49.60)	378(49.61)				
Second infertility	10,643(50.86)	3422(52.87)	1128(50.04)	376(50.40)	384(50.39)				
Infertility causes, n (%)									
Female factor	13,037(62.30)	3914(60.48)	1361(60.84)	471(63.14)	464(60.89)	0.074	0.092	0.087	0.108
Male factor	2534(12.11)	986(15.23)	310(13.86)	121(16.22)	114(14.96)	< 0.001	1.293	< 0.001	< 0.001
Multiple factors	5238(25.03)	1544(23.86)	557(24.90)	151(20.24)	182(23.88)	0.230	0.873	< 0.001	0.356
Unexplained infertility	117(0.56)	28(0.43)	9(0.40)	3(0.40)	2(0.27)	< 0.001	< 0.001	< 0.001	< 0.001
No. of embryos transferred,mean ± SD	1.68 ± 0.51	1.74 ± 0.52	1.72 ± 0.54	1.72 ± 0.56	1.68 ± 0.59	0.244	0.456	0.467	0.674
Development stage of embryos transferred, n (%)						< 0.001	< 0.001	< 0.001	< 0.001
Cleavage stage	8093(38.67)	3074(47.50)	1244(55.61)	433(58.04)	368(48.29)				
Blastocyst	12,833(61.33)	3398(52.50)	993(44.39)	313(41.96)	394(51.71)				
Embryo quality at transfer, n (%)						0.314	< 0.001	< 0.001	< 0.001
Good-quality embryo	20,204(96.55)	6257(96.68)	2132(95.32)	694(92.97)	686(90.04)				
Poor-quality embryo	722(3.45)	215(3.32)	105(4.68)	52(7.03)	76(9.96)				
Year of VF, n (%)						< 0.001	< 0.001	< 0.001	< 0.001
2004.1–2008.12	830(3.97)	242(3.74)	99(4.43)	43(5.76)	59(7.74)				
2009.1–2013.12	4432(21.18)	938(14.49)	412(18.42)	178(23.86)	130(17.06)				
2014.1–2019.8	15,664(74.85)	5292(81.77)	1726(77.16)	525(70.38)	573(75.20)				

*P*1: Group 2 vs. Group 1, *P*2: Group 3 vs. Group 1, *P*3: Group 4 vs. Group 1, *P*4: Group 5 vs. Group 1

**Fig. 1:**
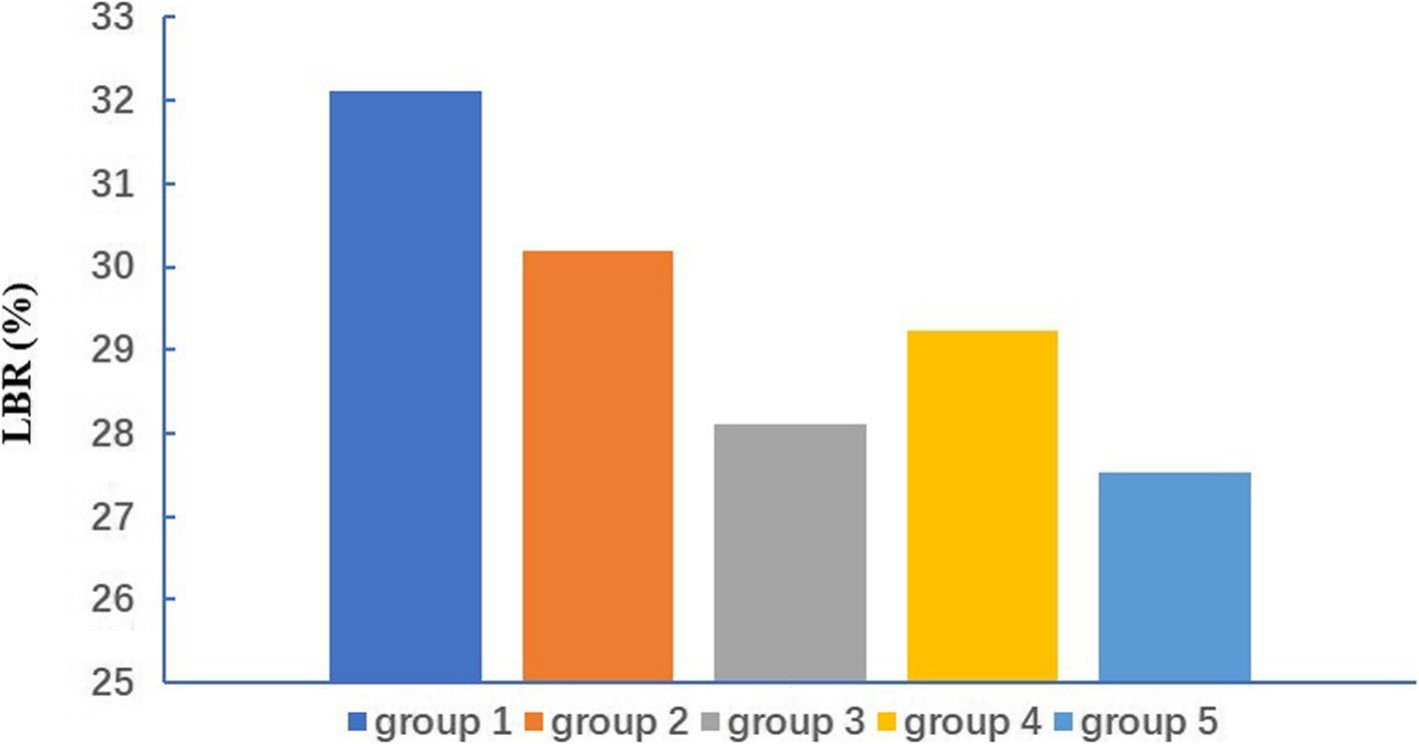
Live birth rate following frozen embryo transfer with different storage times

**Fig. 2:**
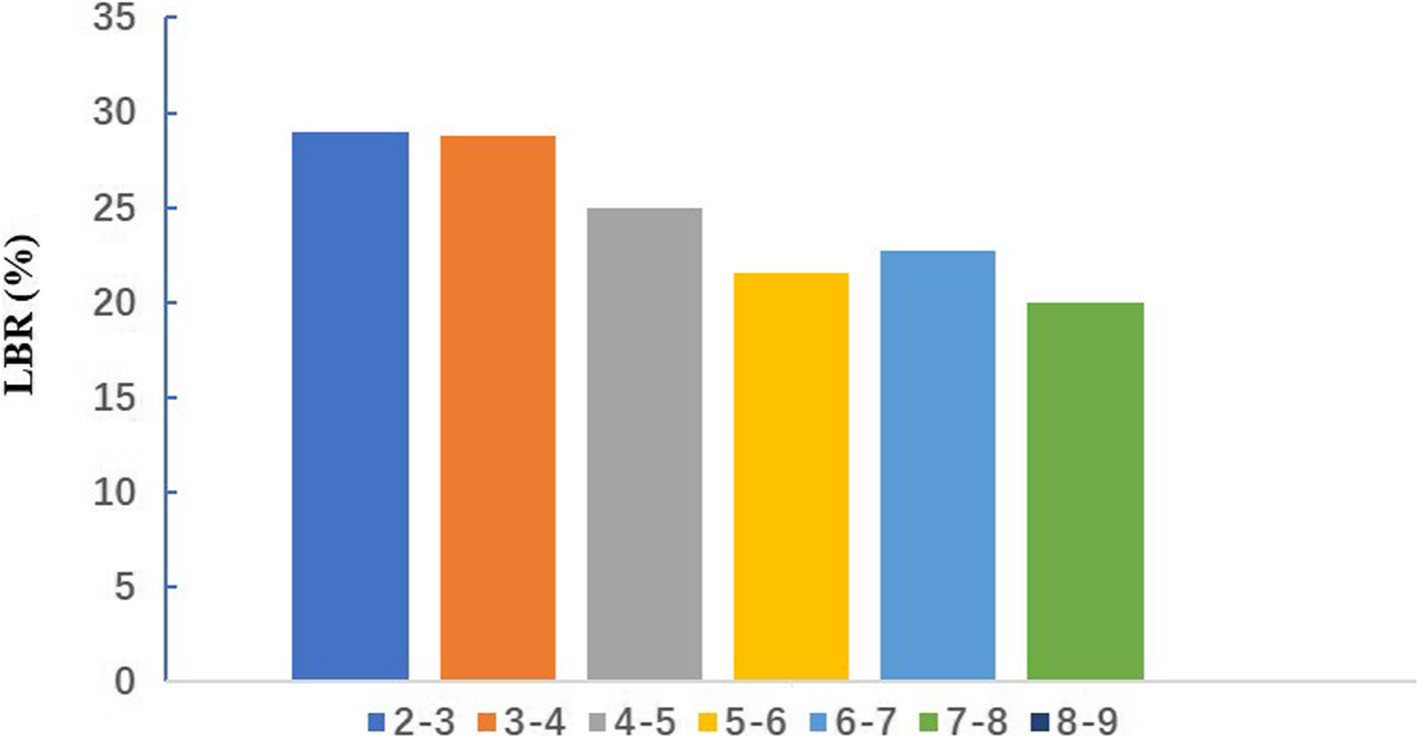
Live birth rate following frozen embryo storage time greater than 2 years

Comparisons of FET pregnancy outcomes among the five groups are summarized in Table 2. Overall, the embryo survival rate decreased significantly as the cryopreservation time increased. The highest and lowest embryo survival rate was 98. 63% in Group 1 and 71.13% in Group 5, respectively (*P* < 0.01). The HCG-positivity (57.85%) and clinical pregnancy rates (55. 26%) were highest in Group 1 (*P* < 0.01). Group 5 recorded the lowest sex ratio (0.96). Overall, the outcomes under consideration were best after a shorter cryopreservation period (Group 1), with a recorded implantation rate of 42.28%, clinical pregnancy per transfer rate of 55.26%, live birth per transfer rate of 32.11%. Figure 1 show the live birth rate following frozen embryo transfer with different storage times. Outcomes of FETs with storage time greater than 2 years showed in Fig. 2 and Table 3.

**Table 2:** Pregnancy outcomes of FETs stratified by the storage time

	1	2	3	4	5	*P*1	*P*2	*P*3	*P*4
survival rate	98.63 (34,853/35338)	94.53 (11,442/12104)	84.62 (3940/4656)	80.75 (1313/1626)	71.13 (1281/1801)	< 0.001	< 0.001	< 0.001	< 0.001
Implantation rate	42.28(14,940/35338)	34.65(3965/11442)	30.18(1189/3940)	32.52(427/1313)	35.21(451/1281)	< 0.001	< 0.001	< 0.001	< 0.001
Positive HCG rate	57.85(12,106/20926)	49.75(3220/6472)	44.43(994/2237)	46.38(346/746)	49.87(380/762)	< 0.001	< 0.001	< 0.001	< 0.001
Clinical Pregnancy rate	55.26(11,563/20926)	47.54(3077/6472)	42.02(940/2237)	43.70(326/746)	47.64(363/762)	< 0.001	< 0.001	< 0.001	< 0.001
Multiple pregnancy rate	30.52(3529/11563)	29.54(909/3077)	27.45(258/940)	30.98(101/326)	25.34(92/363)	0.251	< 0.001	0.682	< 0.001
Live birth rate	32.11(6719/20926)	30.18(1953/6472)	28.12(629/2237)	29.22(218/746)	27.53(210/762)	< 0.001	< 0.001	< 0.001	< 0.001
Ectopic pregnancy rate	1.59(184/11563)	2.08(64/3077)	1.91(18/940)	2.15(7/326)	1.93(7/363)	< 0.001	< 0.001	< 0.001	< 0.001

*P*1: Group 2 vs. Group 1, *P*2: Group 3 vs. Group 1, *P*3: Group 4 vs. Group 1, *P*4: Group 5 vs. Group 1

**Table 3:** Outcomes of FETs with storage time greater than 2 years

Storage time(years)	Storage time(days)	Number of FET cycles	Number of Clinical Pregnancy	Clinical Pregnancy rate	Number of Live birth	Live birth rate	Number of Congenital anomalies
2–3	731–1096	380	191	50.26	110	28.95	1
3–4	1097–1461	205	96	46.83	59	28.78	1
4–5	1462–1826	92	41	44.57	23	25.00	1
5–6	1827–2191	51	22	43.14	11	21.57	0
6–7	2192–2556	22	9	40.91	5	22.73	1
7–8	2557–2921	10	4	40.00	2	20.00	0
8–9	2922–3286	2	0	0.00	0	0.00	0

Comparisons of neonatal outcomes among the five groups are presented in Table 4. No significant between-group differences in mean birth weight, birth height, and rates of congenital conditions were identified. The sex ratios (male/female) were greater than 1 in Groups 1,2,3 and 4, while the proportion of female to male neonates was greater in Groups 5. A total of 178 neonates presented with congenital disorders of the cardiopulmonary system (4 tetralogy of Fallot, 55 congenital heart disease, 5 patent ductus arteriosus), nervous system (5 ventriculomegaly), digestive system (1 congenital esophageal atresia), circulatory system (12 hemangioma), urinary system (8 hypospadias and 2 congenital solitary kidney), musculoskeletal system (16 talipes equinovarus, 3 hip dysplasia, 17 polydactylism), external ear (4 auricle defect), or mouth (24 cleft lip and 22 palate).

**Table 4:** Neonatal outcomes of FETs stratified by the storage time

	1	2	3	4	5	*P*1	*P*2	*P*3	*P*4
Birth sex ratio (male/female)	1.24	1.09	1.33	1.35	0.96	< 0.001	< 0.001	< 0.001	< 0.001
male	5856	1458	475	170	171				
female	4730	1340	358	126	178				
Singleton birth height(cm), mean ± SD	49.01 ± 2.70	49.02 ± 2.58	48.76 ± 2.86	49.39 ± 2.34	49.45 ± 1.91	0.973	0.673	1.082	1.258
Singleton birth weight(kg), mean ± SD	3.03 ± 0.60	2.99 ± 0.61	3.02 ± 0.64	3.04 ± 0.53	3.15 ± 0.38	0.786	0.983	0.819	0.914
Low birth weight (< 2500 g)	19.87(1335/6719)	19.46(380/1953)	18.12(114/629)	15.60(34/218)	15.71(33/210)	0.996	0.351	< 0.001	< 0.001
High birth weight (> 4000 g)	3.66(246/6719)	2.76(54/1953)	3.97(25/629)	1.83(4/218)	3.81(8/210)	< 0.001	0.087	< 0.001	0.068
Congenital anomalies	1.93(130/6719)	1.43(28/1953)	1.91(12/629)	1.83(4/218)	1.91(4/210)	< 0.001	0.293	0.539	0.923

*P*1: Group 2 vs. Group 1, *P*2: Group 3 vs. Group 1, *P*3: Group 4 vs. Group 1, *P*4: Group 5 vs. Group 1

Multivariable logistic regression analysis (Table 5) was performed to determine which variables were significant factors affecting the measured outcomes. The variables included maternal age, maternal BMI, infertility type, infertility causes, stimulation protocols, embryo quality, stage of embryo development, endometrial preparation program, VF years and number of embryos transferred. The result was the same as before.

**Table 5:** Multivariable logistic regression analysis of potential factors associated with outcomes

	Group 2 OR (95% CI)	*P*1 value	Group 3 OR (95% CI)	*P*2 value	Group 4 OR (95% CI)	*P*3 value	Group 5 OR (95% CI)	*P*4 value
Survival rate	0.93 (0.89, 0.98)	0.041	0.86 (0.80, 0.92)	< 0.001	0.82 (0.72, 0.92)	< 0.001	0.71 (0.61, 0.81)	< 0.001
Implantation rat	0.85 (0.78, 0.92)	< 0.001	0.78 (0.71, 0.85)	< 0.001	0.84 (0.80, 0.88)	< 0.001	0.89 (0.84, 0.94)	< 0.001
Positive HCG rate	0.90 (0.85, 0.95)	< 0.001	0.76 (0.69, 0.83)	< 0.001	0.79 (0.73, 0.85)	< 0.001	0.91 (0.86, 0.96)	< 0.001
Clinical Pregnancy rate	0.89 (0.85, 0.93)	< 0.001	0.83 (0.76, 0.91)	< 0.001	0.85 (0.80, 0.91)	< 0.001	0.91 (0.85, 0.97)	< 0.001
Birth sex ratio (male/female)	0.92 (0.87, 0.97)	0.020	1.22 (1.17, 1.27)	0.030	1.17 (1.04, 1.30)	0.039	0.89 (0.82, 0.96)	< 0.001

*P*1: Group 2 vs. Group 1, *P*2: Group 3 vs. Group 1, *P*3: Group 4 vs. Group 1, *P*4: Group 5 vs. Group 1

## Discussion

Concerns have arisen over the safety of prolonged storage time of vitrified embryos worldwide following the wide application of vitrification. It is important to understand the influence of extended storage time on clinical outcomes. Our study demonstrated the safety of using long-stored embryos on neonatal health. As the largest retrospective cohort study, our data suggests that although embryo survival, HCG-positivity, clinical pregnancy rates and live birth rate were significantly reduced, neonatal congenital anomalies were not influenced by storage time. Our advantage is the clinical and laboratory practices remained unchanged throughout the study period, which should minimize the possible confounders associated with outcome. Our study is limited by its retrospective and single-center design.

Earlier studies have shown that after long-term storage, the survival rate and the pregnancy rate of embryos is a trend of decrease after 6–15 months [17]. Testart et al. [18] found increased rates of human embryonic cell death following a cryo-storage period of several months. However, other studies have found that the duration of low-temperature preservation has no significant influence on the quality, survival, implantation capacity, and live birth rates of thawed embryos [19, 20]. Further, Cohen et al. [21] did not identify any deleterious effects related to cryo-storage in their study. Animal studies and theoretical modeling speculate that a frozen mammalian embryo should not be influenced by storage time for several thousand years. 1988, Mazur et al. [22] believe it can be frozen for millennia in liquid nitrogen, the cells can still survive. Because of the enzyme activity in the cell in liquid nitrogen almost completely suppressed, cell processes are at a complete stand still. According to this article, when temperatures below -130 °C, many cells can stability for centuries or millenia. In addition, Glenister et al. [23] exposed frozen mouse embryos to an environment equivalent to 2000 years of cumulative radiation, and their embryos fetal resuscitation rate, developmental potential and reproductive capacity of offspring were not affected, and the rate of offspring variation was not increased. Ma et al. [24] found that a long storage time (less than 8 years) did not influence pregnancy outcomes of FET cycles. Canosa et al. [25] support the safety of long-term cryo-storage of human embryos beyond 12 months. But there are many potential confounders, such as female age at the time of oocyte retrieval, female BMI, infertility type, infertile years, causes of infertility, parity, embryo quality, stage of embryo development, and number of transferred embryos, which can influence the results. Few studies provided these data in the meta-analysis performed by Ma et al. Most data did not adjust for confounders in the meta-analysis performed by Canosa et al. In a word, meta-analysis was conducted using summarized statistics rather than individual data. Acquiring and examining individual data would give a more accurate picture of the dose–response relationship and offer better control of potential residual confounders. Surprisingly, few studies [26, 27] have aimed to address the clinically relevant question of how prolonged periods of storage affect the outcomes of cryopreserved embryos in practice.

Based on the potential impacts freezing methods may have on IVF, embryonic, and perinatal outcomes, VF rather than SF is now routinely used for embryo cryopreservation [4, 28, 29]. Kuang et al. [26] focused on analyzing how storing cryopreserved embryos for two years or less affects pregnancy and neonatal outcomes. However, the perinatal outcomes of embryos stored for longer than two years were not considered. In this study, we examined 31,143 FET cycles with storage times ranging from 30 to 1660 days. In agreement with the findings from the study conducted by Kuang et al. [26], we found that the embryo implantation and pregnancy rate gradually decreased as the storage time increased. However, our results also differed in some respects. For example, Kuang et al. [26] theorized that the duration of embryonic storage would not affect the survival rate after thawing, but our data show a decline in survival rate associated with an extension of storage duration, especially for the group of embryos frozen for more than 2 years (71.13%). During vitrification, embryos are exposed to cold cryoprotectant and directly bonded with liquid nitrogen in an open vitrification system. Prolonged periods of exposure in open vitrification systems may alter the patterns of early embryonic development and influence the post-thaw survival potential.

Pregnancy outcomes may be affected by maternal age at ET and the stage of the embryo being transferred (i.e., cleavage-stage or blastocyst). In our study, the maternal age at ET was lowest and the proportion of transplanted blastocyst-stage embryos was highest in Group 1. Therefore, we identified maternal age at ET and transferal of blastocyst-stage embryos as potential confounders in the determination of pregnancy outcomes in this group. Multivariable logistic regression was performed to explore the effects of storage time on pregnancy outcomes after controlling for the potential confounders. Our results showed that the likelihood of good pregnancy outcomes decreased with the extension of storage durations. Therefore, poor pregnancy outcomes may be caused by prolonged storage rather than maternal age at ET and development stage of embryos.

There were no statistical differences in birth weights and heights among the groups. However, the sex ratio was greater than 1 in Groups 1,2,3 and 4 (*P* < 0.05), while the proportion of female to male neonates was greater in Groups 5. It is known that maternal advanced age and blastocyst-stage embryos significantly increased the proportion of male neonates. After controlling for these confounders, multivariate logistic regression indicated that differences in sex ratio were associated with storage time. In humans, the sex ratio at birth which is known as the secondary sex ratio (SSR), is around 105:100 [30]. Many population-based retrospective study confirms that a specific ART regimen can alter the SSR of babies born through ART was a little higher [31–33]. In fact, previous reports have demonstrated that female embryos are more sensitive to than males to the vitrification process [34]. We hypothesized that the tolerance of female embryos to low temperatures decreased significantly with longer storage time in liquid nitrogen. Group 5 had a sex ratio less than 1. This possibly because the mothers were the oldest in the group at the time of thawing and the embryos were of poor quality, which could lead to an increased proportion of female offspring.

## Conclusions

In summary, we hypothesized that prolonged durations of cryopreservation may be associated with a decline in embryo survival rates. We found that the clinical pregnancy rate was higher following short-term storage (i.e., no more than three months). Therefore, although long-term cryopreservation does not affect neonatal health, cryopreserved embryos should be recovered as soon as possible to support successful implantation. However, when conditions preclude short-term storage, long-term storage is clinically feasible.

## Data Availability

The data sets in this study cannot be publicly available because of involving the patient privacy but are available from the corresponding author on reasonable request.
